# Development of a Smart Chair Sensors System and Classification of Sitting Postures with Deep Learning Algorithms

**DOI:** 10.3390/s22155585

**Published:** 2022-07-26

**Authors:** Taraneh Aminosharieh Najafi, Antonio Abramo, Kyandoghere Kyamakya, Antonio Affanni

**Affiliations:** 1 Polytechnic Department of Engineering and Architecture, University of Udine, Via delle Scienze 206, 33100 Udine, Italy; aminoshariehnajafi.taraneh@spes.uniud.it (T.A.N.); antonio.abramo@uniud.it (A.A.); antonio.affanni@uniud.it (A.A.); 2 Institute for Smart Systems Technologies, Alpen-Adria University, 9020 Klagenfurt, Austria

**Keywords:** smart chair, pressure sensors, deep learning models, sitting postures classification

## Abstract

Nowadays in modern societies, a sedentary lifestyle is almost inevitable for a majority of the population. Long hours of sitting, especially in wrong postures, may result in health complications. A smart chair with the capability to identify sitting postures can help reduce health risks induced by a modern lifestyle. This paper presents the design, realization and evaluation of a new smart chair sensors system capable of sitting postures identification. The system consists of eight pressure sensors placed on the chair’s sitting cushion and the backrest. A signal acquisition board was designed from scratch to acquire data generated by the pressure sensors and transmit them via a Wi-Fi network to a purposely developed graphical user interface which monitors and stores the acquired sensors’ data on a computer. The designed system was tested by means of an extensive sitting experiment involving 40 subjects, and from the acquired data, the classification of the respective sitting postures out of eight possible postures was performed. Hereby, the performance of seven deep-learning algorithms was assessed. The best accuracy of 91.68% was achieved by an echo memory network model. The designed smart chair sensors system is simple and versatile, low cost and accurate, and it can easily be deployed in several smart chair environments, both for public and private contexts.

## 1. Introduction

Scientific and technological advancements that have been made during the last decades have radically changed the lifestyle of most of the people living in post-industrial countries. Thanks to the modernization of industries and cities, the need for human physical activity in daily tasks has been reduced significantly. Nowadays, machines have taken charge of almost all heavy occupations. As a result, jobs have mainly to do with office tasks in which personal soft skills are mostly required as opposed to hard ones and sitting still in front of computer screens for extended periods of time is the more frequent habit. Commuting is generally performed using personal vehicles or public transportation systems. Houses are structured to provide maximum comfort, obtained by means of modern facilities that require moderate or no physical activities. After many sedentary working hours, television and social media occupy large numbers of people, who once again remain seated in front of their screens. On the other hand, inactivity aiming at preserving the vital energy needed for survival and for food search is the preferred genetic choice [1]. Indeed, our hunter-gatherer ancestors were considerably more active than we are as individuals in modern societies [2]. Nevertheless, they had long sedentary hours during the day as well, but opposite to what happens in modern times on modern chair designs, their sitting postures were engaging more muscular activities than today [3]. Consequences of such habits are that the current sedentary lifestyle is known to increase the risk of several chronic health conditions [4], such as cardiovascular diseases [5], obesity [6], type 2 diabetes [7,8], colon cancer [9,10], osteoporosis [11], and depressive illnesses [12]. Furthermore, thanks to high levels of automation and the integration of Artificial Intelligence (AI) in many aspects of our daily life, future lifestyles with increased sedentary attitudes are easily predictable for the majority of individuals. Therefore, it is very important to investigate and implement methods able to reduce the negative effects of this unavoidable sedentary lifestyle.

In response to the side effects of a sedentary life style, recently, sitting posture monitoring and corrective applications have received much attention among researchers. Several kinds of smart chairs have been developed and experimented using various methods, including image processing [13,14] and chairs’ sensor data classification, using various sensor types such as single pressure sensors [15,16,17,18], pressure distribution sensors [16,19,20,21], textile sensors [22,23], accelerometers [24], and combined sensors [25,26], just to name a few. For example, in [20] the authors adopted an 8 × 8 pressure sensor array positioned on the sitting cushion of a chair. They employed an Artificial Neural Network (ANN) model to classify eight sitting postures. The model achieved a classification accuracy of 92.2% in the subject-independent case by using the data collected from eight subjects as the training set and the data collected from another eight subjects as the test set. In [19], pressure mapping sensors were mounted on the surface of the sitting cushion and backrest of a chair, and a Principal Components Analysis (PCA) model was used to identify pattern similarity and to classify 10 sitting postures. This study achieved a sitting postures classification accuracy of 96% and 79% in the subject-dependent and subject-independent cases, respectively. In [16], the authors presented an optimization model for sensor positioning to reduce the number of pressure sensors to be deployed. Using a logistic regression classifier, they could achieve a classification accuracy of 87% for 10 postures with 31 pressure sensors, and of 78% with 19 pressure sensors in subject-independent cases. In [23], the design of a textile sensor, named eCushion, was presented. The system included a 16 × 16 sensor textile array, a signal acquisition unit based on an Arduino board equipped with Bluetooth for data transmission, and a smartphone featuring a custom application for computation and data monitoring. The experimental results for seven postures classification showed a recognition rate of 92% in the subject-dependent case, and 79% in the subject-independent case. In [25], the authors designed a mixed sensors system combining six pressure sensors and six infrared sensors. This combination allows the user to perform pressure measurements on the sitting cushion by means of the pressure sensors and the backrest to measure back distance by means of the reflective ones. The authors employed a k-Nearest Neighbor (KNN) algorithm to classify 11 sitting postures, achieving 92% of classification accuracy in the subject-independent case. Finally in [15], a smart Internet of Things (IoT) system for sitting posture detection was proposed. It was based on the installation of six force sensors, communicating with a mobile application. In this design, several chairs were equipped with the six force sensors and a NodeMCU board. Resistance measurements read by the sensors were sent to a cloud-based platform, where a real-time algorithm was running, providing postures classification in three categories, green (correct), orange (wrong) or red (unhealthy), defined by means of three experimentally determined separation thresholds. A smartphone application was monitoring the postures classification results for the user in real-time.

This review of the smart chair studies for sitting postures classification obtained with various methods and sensing devices shows that in most cases a high classification rate was achieved when a large number of sensors were deployed (≥30) [16,19,20,23], or when sensors fusion was adopted [25,26]. In some cases, results were not validated with k-fold cross-validation methods [18,20,21,23,26]. In many cases, the proposed systems were evaluated by means of experimental setups involving the participation of few subjects (≤10) [18,23,26], as their results could be affected by high variance, thereby producing poor generalization performances [27].

This paper presents the achievement of our three main objectives for this project: (1) design a new, low cost and simple smart chair sensors system that has the advantage of being at the same time robust, versatile, accurate, and easy to setup; (2) optimize the number and position of the sensors to reach a high classification accuracy; and (3) identify the best deep learning model for our designed smart chair.

To achieve the mentioned objectives, we performed the following steps. (1) We designed the smart chair sensors system employing eight low-cost resistive pressure sensors which were placed on the sitting cushion and on the backrest of a chair. (2) The sensor signals were acquired by a purposely designed data acquisition unit. In contrast to almost all the aforementioned studies that adopted a commercial product such as Arduino, we designed the acquisition system and realized a Printed Circuit Board (PCB) specifically for the smart chair project. The designed PCB presents several advantages with respect to Arduino-based boards, since we could customize the components’ choice according to our specific needs. A first advantage is that the chosen Digital Signal Processor (DSP) has an on-board 12-bit Analog to Digital Converter (ADC) with 16 analog inputs (DSPIC30F6014A from Microchip), while normally Arduino features a 10-bit ADC. This choice increased the measurement resolution by a factor of four, allowing us to resolve 0.00025 kg force acting on each sensor. A second advantage with respect to Arduino-based boards is that we can integrate the entire electronic circuitry on a unique PCB, thus eliminating any auxiliary boards (e.g., Wi-Fi module) that usually must be externally wired to commercial boards. (3) We obtained detailed characterization of the analog front-end of the setup by performing linearity, repeatability, and bandwidth analysis of the sensors system. (4) The designed board was placed on the back of the chair, hosted in a purposely designed 3D-printed enclosure. It is battery powered and can transmit data to a laptop via Wi-Fi continuously up to 30 h without recharging. Therefore, the system is wireless, thus avoiding the need for cumbersome cabling. (5) We developed a Graphical User Interface (GUI) application on a laptop. It receives data and stores them locally, while plotting them in real time on the screen. (6) An experiment has been set up, and a dataset has been collected from 40 individuals sitting in eight different postures. (7) The acquired dataset was employed to train and test seven different deep learning models. (8) To find the most suitable model for our application, the average classification accuracies of all models, obtained by k-fold cross validation, were compared. As it will be shown, the Echo Memory Network (EMN) model scored the best average classification accuracy of 91.68%. The choice of the sensors type, their number, and positions in combination with the custom designed acquisition board that transmits the acquired data without wires to the developed GUI on a computer are the main innovative aspects of the designed smart chair, which produced relatively high classification accuracy.

The paper is organized as follows. Section 2 describes the design of the smart chair sensors system (hardware and software components), the experimental setup used to acquire the sitting postures dataset (with preprocessing steps), and the Deep Learning (DL) models’ architecture and hyperparameters. Section 3 shows the experimental results obtained from the characterization of the analog front-end of the sensors system and the results achieved during the postures classification experiment, including result comparison among the seven DL models. Finally, Section 4 presents the discussion on various sitting patterns and the related challenges, the computational costs of each DL model, and a comparison between the results of our study and other similar ones. Finally, Section 5 draws the conclusions of the paper.

## 2. Materials and Methods

This section is organized in three parts: the first part describes the design of the developed smart chair sensors system as performed according to the required technical specifications; the second part describes the experimental setup and the procedure followed to acquire data from the 40 participants sitting in eight different sitting postures; the third one describes the architecture of the seven DL models employed for the classification of the eight sitting postures.

**Figure 1:**
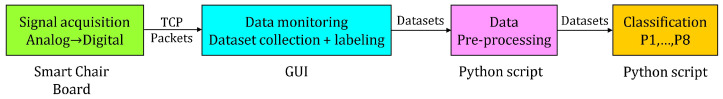
The structure of the proposed Smart chair posture recognition system.

The general structure of the proposed smart chair posture recognition system is presented in Figure 1. The smart chair sensors system board (shown as the green box in the figure) has the duty to acquire signals from the chair’s sensors, convert them into digital data, and transmit them to the computer by means of a TCP link. These operations are described in Section 2.1.1 and Section 2.1.2, respectively. The designed GUI (shown as the turquoise box in the figure) has the duty to monitor the received data, collect datasets, label them, and store them as text files. The GUI is explained in detail in Section 2.1.3. Data preprocessing steps (shown as the pink box in the figure) and classification of the eight sitting postures P1, *…*, P8 (shown as the yellow box in the figure) were coded in Python scripts and are described in detail in Section 2.2.2 and Section 2.3, respectively. Sitting postures are described in Section 2.2.1.

### 2.1. Smart Chair Sensors System

**Figure 2:**
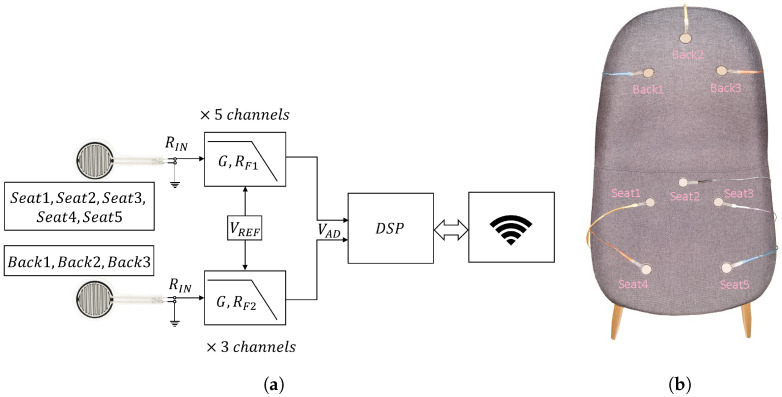
The Smart Chair sensors system: (**a**) block diagram of the sensors system circuit. Analog front-end has seat and backrest circuits that convert the sensors signals $R_{IN}$ to voltage. DSP converts the analog voltage signals to digital data packets. Wi-Fi module transmits the TCP packets to the computer; (**b**) top view of the chair equipped with labeled FSR sensors.

The developed sensors system (see Figure 2a for the block diagram) evaluates the user’s body pressure on the sensorized chair by means of eight Force Sensing Resistors (FSR); five of them were placed on the sitting cushion and three on the backrest of the chair, as shown in Figure 2b. The location of each sensor was adjusted to favor the classification of the eight sitting postures. We verified that the conductance of the FSR sensors changes almost linearly with the applied pressure, i.e., the resistance of the sensors decreases by increasing the applied pressure. A sensors system circuit was designed to: (1) convert the conductance of each sensor into a voltage; (2) condition the signals by filtering and amplification according to the specifications described in Section 2.1.1; (3) process the signals by a DSP; (4) transmit the data by a Wi-Fi module to a laptop where a custom application GUI, as described in Section 2.1.3, was developed for data storage and real-time monitoring.

**Figure 3:**
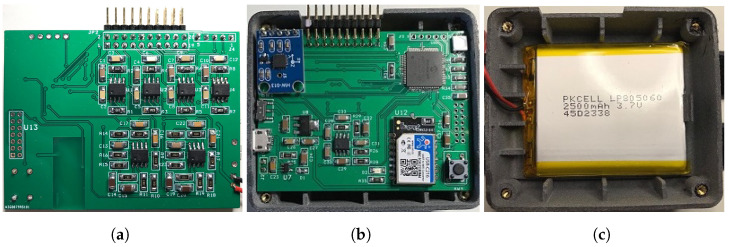
The Smart Chair sensors system circuit, battery, and enclosure: (**a**) circuit top layer including the analog front-end circuits; (**b**) circuit bottom layer (PCB inserted into the enclosure) including the power supply, DSP, Wi-Fi module circuits; (**c**) the enclosure hosting the battery.

The sensors system is powered by a single 2500 mAh Lithium-Polymer (LiPo) rechargeable cell battery (see Figure 3c). With a multimeter, we experimentally measured the current consumption of the entire board during continuous transmission, obtaining as a result 85 mA; the main contribution of current consumption is due to the Wi-Fi module whose consumption is 70 mA. Thus, the LiPo cell allows for ≈30 h of continuous transmission without battery recharging. The choice of a battery-operated system allows us to easily use the chair without the need of power line plugs or wires, with an advantage of usability with respect to the existing literature (e.g., [20,23]). A buck DC–DC converter (not shown in Figure 2a) provides a +3.3 V supply voltage for the entire circuit. A reference voltage $V_{REF}$ = 100 mV, required for the Op-Amps implementing low-pass active filters and the conductance to voltage conversion, is generated using a linear voltage reference and routed to the non-inverting input of the Op-Amps.

For the circuit assembly, a two-layer PCB was printed and populated on both sides. Its top layer, shown in Figure 3a, holds the analog front-end, while the bottom one, shown in Figure 3b, holds the power supply, the reference voltage $V_{REF}$ generation, and the Wi-Fi module. The PCB and the battery were accommodated in a custom-designed 3D-printed enclosure, shown in Figure 3b,c. The external dimensions of the circuit and of the enclosure are 70 × 53 ${\mathrm{mm}}^{2}$ and 75 × 65 × 34 ${\mathrm{mm}}^{3}$, respectively.

#### 2.1.1. The Analog Section

Referring to Figure 2a, the sensors’ readout signals $R_{IN}$ are connected to a conductance to voltage converter circuit with a first-order low pass behavior. The minimum conductance $G_{IN}\equiv 1/R_{IN}$ of the sensors is nearly zero when no pressure is applied and increases with pressure towards a maximum value $G_{IN,MAX}$. Indeed, sensors on the sitting cushion are subject to a pressure that is significantly higher than that activating the backrest sensors. We experimentally determined the maximum resistance for the two cases, obtaining $1/G_{IN,MAX}\equiv R_{IN,MIN}$ = 12 kΩ for the sitting cushion case and $R_{IN,MIN}$ = 17 kΩ for the backrest one. To obtain the full scale of the measurement with the minimum resistances of the sensors, we calculated the feedback resistor of the filters to be $R_{F1}$ = 390 kΩ for the sitting cushion circuits and $R_{F2}$ = 560 kΩ for backrest circuits. The transfer function of the analog section for each channel is:

(1) $$V_{AD}=V_{REF}\cdot \left(1+\frac{R_{F}}{1+j\omega \;\tau_{LP}}\cdot G_{IN}\right),$$

where ω is the angular frequency. Designing $\tau_{LP}$ = 32 ms, the analog section behaves as a low pass conductance to voltage converter, having a cutoff frequency of 5 Hz.

#### 2.1.2. ADC, DSP, and Data Transmission

The signals $V_{AD}$ are routed to the analog inputs of the DSP (DSPIC30F6014A from Microchip), featuring an on-board 12-bit ADC and operating at the peak rate of 8 MIPS. The DSP receives the analog signals and converts them into 12-bit numbers operating with a 10 Hz sampling rate. After conversions, the DSP assembles the data packets and sends them through an on-board Universal Asynchronous Receiver Transmitter (UART) to a low-power Wi-Fi module (USR-C216), which sends them to a computer via TCP protocol.

#### 2.1.3. The Software Description

**Figure 4:**
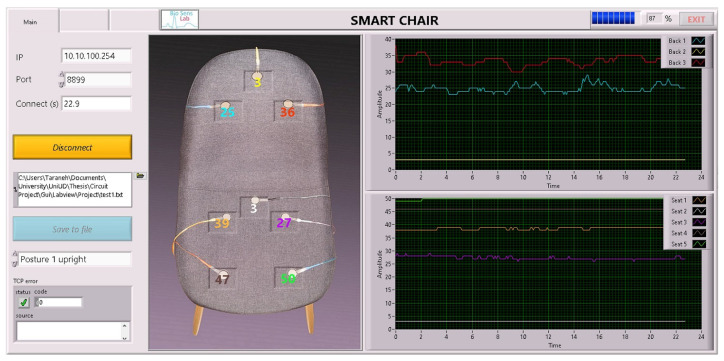
GUI front panel developed in LabVIEW™ environment for data acquisition and real-time plot. The sensor signals evolution in time are plotted on the graphs; the top and bottom graphs show the back and seat sensor signals, respectively. The real time sensor values are shown on a top view image of the chair in colored numbers. Controls are located on the left side of the front panel.

We developed a GUI using LabVIEW™ from National Instruments. The front panel of the GUI is shown in Figure 4. The GUI is designed to acquire the transmitted data from the chair’s Wi-Fi module, visualize them in real-time, and store them locally on the computer.

To monitor the real-time data from the chair sensors, two graphs are provided as shown at the right side of Figure 4: the top chart plots the time behavior of the three signals coming from the backrest sensors, while the bottom chart plots the time behavior of the five sitting cushion sensors. Signal values were scaled to be always in the dimensionless range [0, 100]%, where 100% and 0% indicate the maximum and minimum pressure values, hence minimum and maximum sensor resistance, respectively. On the left side of the GUI, in addition to the chair and sensors sketch, few controls were added, such as the IP address, the port of the TCP communication protocol, and the path and name of the store file. In addition, a selectable list of different postures to test was implemented to tag the present acquisition with the corresponding label. The front panel was furnished with a button to connect/disconnect the data acquisition and monitoring. Finally, possible communication errors were visualized in an appropriate TCP error box.

### 2.2. Experimental Setup

The designed sensors system described above was mounted on the chair shown in Figure 2b. Three sensors (tagged Back1, Back2, Back3) were mounted on the backrest, while five sensors (tagged Seat1, Seat2, Seat3, Seat4, Seat5) were mounted on the sitting cushion. The 3D-printed enclosure containing the embedded electronics was mounted on the rear of the chair. Since the board is battery-operated and data transmission is wireless, there are no cables extending out of the chair which consequently can be freely moved around, a relevant feature for practical applications.

#### 2.2.1. Sitting Postures

Sitting and standing can occur in various positions, named postures. A posture is the preferred bio-mechanical position of the body in space. The muscular system works together with the skeletal system to keep the body in balance during either dynamic movements or static conditions [28].

**Figure 5:**
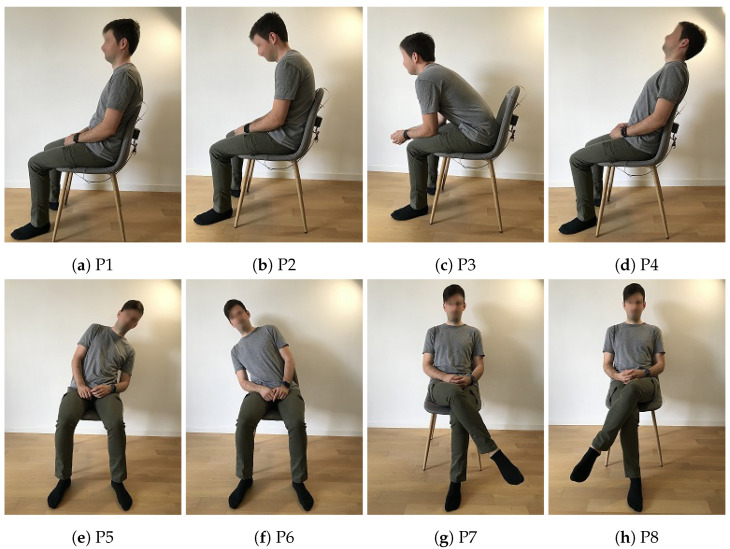
The eight sitting postures: (**a**) P1: upright; (**b**) P2: slouching; (**c**) P3: bending forward; (**d**) P4: bending backwards; (**e**) P5: bending left; (**f**) P6: bending right; (**g**) P7: right leg above; (**h**) P8: left leg above.

Eight sitting postures were practiced during the setup experiment in which 40 volunteers participated (28 males, 12 females, age 39 ± 17). Each subject sat still on the smart chair assuming in turn, all eight postures, maintaining each one for 60 s and repeating each posture three times. The first posture, P1 of Figure 5a, represents the sitting position upright with straight back and symmetric limbs. Generally, this is the recommended posture when sitting on a chair, while the second posture, P2 of Figure 5b, represents slouching with curved back, inclining the head forward and lowering the shoulders. This posture and its variations are very common among office employees. In contrast to P1, in this posture, the functionality of internal organs, such as heart, lungs and digestive system, may in the long term become negatively impacted, and lead to backache and pain [29,30]. The third posture, P3 of Figure 5c, shows a forward bending, with straight back and symmetric limbs, while P4 of Figure 5d is similar to P3, but the bending direction is backward. The fifth and sixth postures, P5 and P6 of Figure 5e,f, respectively, represent the bending of the torso toward the left and right sides, respectively, while the legs remain symmetrical and the feet are flat to the ground. Finally, the last two postures, P7 and P8 of Figure 5g,h, are the case where the right leg crosses over the left one and vice versa, the torso remaining upright and erect.

As a matter of fact, each posture creates a different weight distribution on the eight sensors of the chair. Indeed, several factors (such as weight, height, body shape, muscle strength, etc.) influence the way someone sits on a chair, but in general, the distribution of the weights obtained for a given posture by different subjects is expected to show a similar pattern.

#### 2.2.2. Data Acquisition and Preprocessing

Before sitting on the smart chair, the volunteers were informed about the purposes of the study, the procedures of the test, and data anonymization performed for privacy enforcement. Each session of the experiment was conducted in three steps: (1) the subject was invited to sit on the chair with empty pockets to keep the symmetry of the weight distribution on the sensors (the presence of wallet or smartphone in the back pockets could alter the pressure distribution); (2) all sitting postures were explained and demonstrated to the subject; and (3) the subject was guided to sit in each posture in a natural and comfortable position and to keep it for the 60 s, of the data acquisition phase. As said, each of the eight postures was repeated three times. Thus, 24 datasets were generated for each subject. Since the sampling frequency was 10 Hz, each dataset contained approximately 600 samples. Given the 40 subjects, a total of 960 datasets were generated, for a grand total of $5.76\times 10^{5}$ samples. Each dataset contains the acquisition time for each sample, the eight readouts of the eight sensors, and the indication of the posture type annotated by the designed GUI.

To standardize the datasets in view of the DL analysis, a simple Python code was developed to preprocess each file. The first and the last 10 samples (i.e., the first and last seconds of acquisition) were discarded to remove possible transient behaviors due to the sitting and get up actions of the subject, or because the posture changed. Consequently, each dataset was reduced to 580 samples for a new grand total of $5.568\times 10^{5}$ samples, an appropriate procedure that was performed without losing generality.

To reduce the inherent noise contributions, artefacts, and spurious values possibly due to the subjects’ movements during signal acquisition were smoothed using a Savitzky–Golay filter from the scipy.signal library, whose window length and polynomial order were set to 301 and 3, respectively.

Since the sensor values stored by the GUI were designed to be in the percentage range [0, 100], normalization to unity was performed to bound all values within the [0, 1] real interval.

### 2.3. Sitting Postures Classifications

To classify the eight sitting postures with the highest accuracy, we tried seven different DL models taken from three main categories: (1) feedforward artificial neural networks, and we adopted Multilayer Perceptron (MLP); (2) Convolutional Neural Networks (CNN); and (3) Recurrent Neural Networks (RNN), where we adopted a Long Short-Term Memory (LSTM), a bidirectional LSTM (BDLSTM), a CNN-LSTM (CNLSTM), a convolutional LSTM (CVLSTM), and an Echo Memory Network (EMN). All models were trained and tested with the very same datasets collected during the experiment. As mentioned in Section 2.2.2, datasets have two dimensions: the first dimension is the number of samples (580), the second dimension is the number of sensor signals (8). Since CNN and RNN models require three-dimensional input data, we converted the datasets to frames with 10 samples of 58 time steps of 8 sensors signals. Consequently, the input dimensions of the models were set to [samples, time steps, and features] whose values were specified as being [10, 58, 8]. The results of the comparison among the different models are shown in Section 3.2.

In feedforward ANNs, or equivalently MLPs, information flows acyclically from the input neurons’ layer to the output one, traversing an arbitrary number of so-called hidden layers. The idea behind MLPs is that the complexity of the nonlinear approximation required to classify arbitrary functional behaviors can be tackled by a supervised learning procedure, whose core is based on the use of the notorious back-propagation algorithm which, formalized in a divide and conquer structure, enables the deployment of very deep networks, as opposed to a wide single hidden layer one that can be solved in a reasonable amount of time. In this way, both classification and regression problems can be successfully tackled with reasonable computing resources [31]. Besides being acyclic, the MLP model is in general fully connected, which means that each neuron of a given layer receives inputs from all the neurons of the preceding one and provides output to all the neurons of the subsequent one.

**Figure 6:**
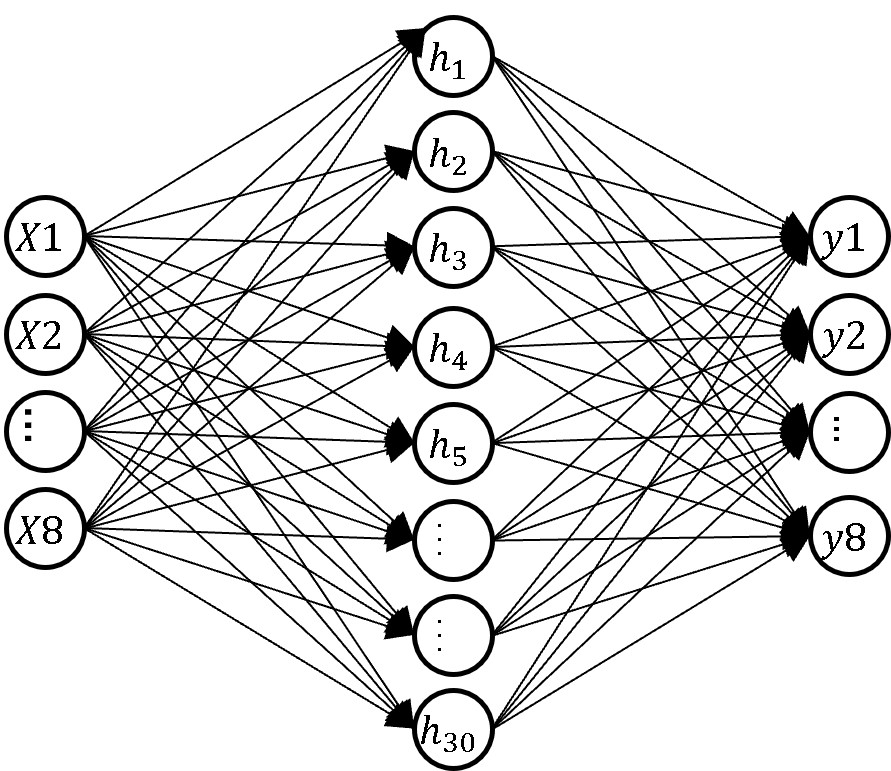
The adopted MLP model architecture. It consists of an input layer with 8 neurons for 8 sensors signals; a hidden layer with 30 neurons, and an output layer with 8 neurons for 8 postures classification.

Nevertheless, we adopted an MLP model with a single hidden layer of 30 neurons, as shown in Figure 6. We used the Python scikit-learn library to implement the learn and test procedures. A rectified linear unit (ReLU f(x)=max(0,x) [32]) was used as activation function, while ADAM [33] was used as stochastic gradient-based optimizer, where the regularization parameter (L2 penalty) introduced to limit the data overfitting was set to 0.7, and the maximum number of iterations (training epochs) was set to 100. The input and output layers consisted of eight neurons each, corresponding to the eight input sensors and to the eight postures, respectively, and we used the softmax activation function for the classification. The Cross-Entropy (log loss) was adopted as the loss function for all the models.

**Figure 7:**
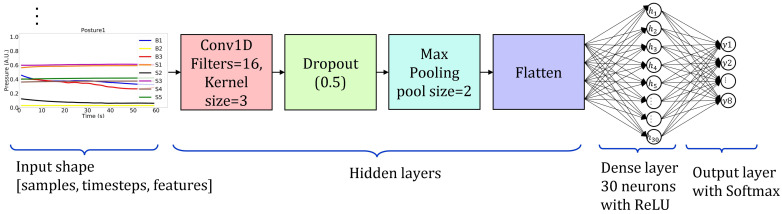
The adopted CNN model architecture. Input has a 3D shape. Hidden layers are Conv1D with 16 filters, dropout, Max pooling, flatten, and dense layer with 30 neurons. The output layer has 8 neurons for 8 postures classification.

CNNs are specially designed to process data with grid-like topology. The kind of data that can be fed to CNNs is either 1D grids, as in the case of time series where signal sampling produces a data stream where neighboring points (with a proper definition of the neighborhood) are correlated in time, or 2D grids, as in the case of digital images, where instead, spatial correlation occurs among neighboring pixels. CNNs apply successive convolution filters to input, performed using proper numerical kernels (filters). Assuming a 2D problem, hence matrix kernels, these filters are usually significantly smaller than input matrices. Consequently, the CNN elaboration aims at extracting the most meaningful features of the input matrix, thus preventing the overfitting problem with the advantage of lower memory requirements for parameter storage [31]. We implemented a CNN model using the Keras library and designed it with a 1D convolutional layer of 16 filter kernels with size of 3, followed by a dropout layer for overfitting mitigation and a MaxPooling1D layer for the downsampling. Then, a flattening layer was added and a further fully connected layer of 30 neurons with L2 = 0.7 and ReLU activation for information restoration was stacked, followed by an additional fully connected output layer comprising eight neurons with softmax activation functions. Figure 7 shows the architecture of the CNN model as described above. The model was fitted and validated in 100 epochs with batch size of 64 input samples.

RNNs are neural networks specialized to process sequential data [31]. They are networks with memory that allows the network to remember the time sequence of the inputs. The memory is fed by recurrent connections, allowing the hidden cells to have access to their previous outputs [34].

As mentioned before, we trained and tested five different RNN models: LSTM, BDLSTM, CNLSTM, CVLSTM, and EMN.

**Figure 8:**
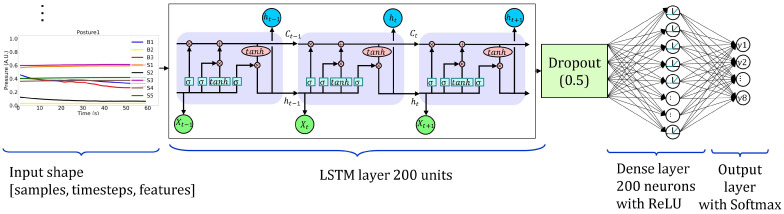
The adopted LSTM model architecture. Input has a 3D shape, hidden layers are LSTM with 200 units, dropout, and a dense layer with 200 neurons. The output layer has 8 neurons for 8 posture classification.

LSTM networks overcome the vanishing gradient problem typical of RNNs by avoiding the long-term dependency problem. LSTMs, instead of using neurons, possess memory blocks that are connected to layers by gates (input, output, and forget) that are included to manage the block state and output [35,36,37]. We implemented an LSTM model using the Keras library, featuring an LSTM layer of 200 units followed by a dropout layer and a fully connected layer of 200 neurons using L2 = 0.7 and ReLU activation functions. The output was obtained with a single, fully connected layer containing eight neurons with softmax activation functions. Figure 8 shows the architecture of the LSTM model. Moreover, this model was fitted and validated in 100 epochs with input batch size of 64 samples.

The BDLSTM [38] combines an LSTM processing data forward in time starting from the beginning of the sequence with one additional LSTM which instead processes backwards in time starting from the end of the sequence [31]. We employed a BDLSTM model present in Keras, configured with a bidirectional wrapper comprising an LSTM layer of 200 units, followed by a dropout layer and a fully connected layer of 200 neurons with L2 = 0.7 and ReLU activation functions. The fully connected output layer contained eight neurons with softmax activation functions. This model was also fitted and validated using 100 epochs with batch sizes of 64 samples.

The CNLSTM model [39] utilizes a CNN to extract important features from the input data, which is combined with an LSTM for sequence memorization. Again, we employed the CNLSTM model present in the Keras library with two time distributed wrapper Layers for the time optimization, including a convolution layer of 64 filters with kernel size 1×3 and ReLU activation function, followed by three additional time distributed wrapper layers comprising a dropout layer, a pooling layer for down sampling, and a flattening layer, respectively. An LSTM layer of 200 units was then added, followed by a dropout layer, and by a fully connected layer for reconstruction made of 200 neurons with L2 = 0.7 and ReLU activation function. The output was then obtained by means of a fully connected layer featuring eight neurons and softmax activation functions. As the previous ones, the model was fitted and validated in 100 epochs with batch size of 64.

Ths CVLSTM [40] uses convolutions to provide inputs to an LSTM. We adopted the CVLSTM model included in the Keras library, in particular its ConvLSTM2D module which is similar to an LSTM but with a convolutional input and feedback transitions. This layer was composed of 16 filters with kernel size 1 × 3, followed by a dropout layer, a flattening layer, and a fully connected layer of 200 neurons with L2 = 0.7 and ReLU activation functions. The output layer was obtained with a fully connected layer of eight neurons with softmax activation functions. Once more, the model was fitted and validated in 100 epochs with batch sizes of 64 samples.

**Figure 9:**
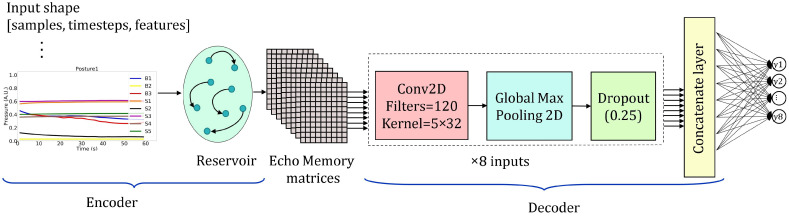
The adopted EMN model architecture. Input has 3D shape, hidden layers consists of an encoder and a decoder. The encoder converts the 3D inputs to echo memory matrices; the decoder consists of the fusion of the features extracted from the 8 sensors signals matrices by CNN2D layer, together with the max pooling and dropout layers. The output layer has 8 neurons for 8 postures classification.

Finally, the EMN [41] model, designed specifically for time series classification, combines Echo State Network (ESN) and CNN models, exploiting both their peculiar characteristics. EMN is composed by encoding and decoding steps. At the encoding step, memory matrices are created by projecting each input time series frame onto a high-dimensional reservoir state space. At the decoding step, instead, the memory matrices are decoded by CNN convolutional and pooling layers. The full description of the model can be found in [41]. We employed the EMN model with a reservoir layer as encoding step, which produced the echo memory matrices from the input time series. As per the decoding step, we created eight inputs using the memory matrices of the eight sensors signals. Each input was generated stacking 2D convolutional layer featuring 120 filters and kernel size of 5 × 32, followed by a 2D pooling layer and a dropout one. Then, we implemented data fusion of all the pooled features using a concatenation layer, followed by a fully connected output layer made of eight neurons with L2 = 0.7 and softmax activation functions. In this case, the model was fitted and validated in 100 epochs with batch size of 25. Figure 9 shows the architecture of the EMN model.

## 3. Experimental Results

In this section, we present the experimental results organizing the discussion in two parts. The first part describes the results obtained from the metrological characterization of the sensor analog front-end. The second part, instead, describes the results on sitting postures classification and compares the average classification accuracy of the seven adopted DL models.

### 3.1. Analog Front-End Characterization

As described in Section 2.1.1, we set by design the minimum resistance of the sensors to be $R_{IN,MIN}$ = 12 kΩ, corresponding to a load on each sensor of approximately 1 kg. We made this choice, estimating the pressure on a sensor with area 5 cm${}^{2}$ as produced by an overweight person of (≈180 kg) sitting on a surface of ≈900 cm${}^{2}$. To characterize the linearity of the analog front-end, we developed a test bench where a pressure sensor was connected to the circuit board and where we measured the output voltage it was producing varying the weight acting on the sensor itself. The weight range we adopted was [0–950] g, which we spanned in *M* = 20 steps, measuring the applied weight at each step with a precision scale whose accuracy was much better than the sensor one. At each weight step *i*, we acquired j=1…N voltage samples $V_{AD,ij}$ (we also chose N=20) to perform type A estimation of the uncertainty:

(2) $$u_{A}\left(V_{AD,i}\right)=\sqrt{\frac{1}{N\times (N-1)}\sum_{j=1}^{N}{\left(V_{AD,ij}-\bar{V_{AD,i}}\right)}^{2}}$$

The voltages were measured using a 6½ digits multimeter HP34401A [42].

We combined the type A estimation of the uncertainty with type B estimation, $u_{B}\left(V_{AD,i}\right)$, (available from the multimeter), following that prescribed by the Guide to the Expression of Uncertainty in Measurement (GUM) [43], obtaining:

(3) $$u_{C}\left(V_{AD,i}\right)=\sqrt{u_{A}{\left(V_{AD,i}\right)}^{2}+u_{B}{\left(V_{AD,i}\right)}^{2}}$$

From the 20×20 matrix of voltages acquisitions, we obtained the mean value of each step and its combined uncertainty. To perform linearity analysis, we used the least squares method using the balance measurements as weight vector $W_{i}$ and the computed sample means $V_{AD,i}$. The resulting gain *G* thus reads:

(4) $$G=\frac{\langle W⊙\bar{V_{AD}}\rangle -\langle W\rangle \langle \bar{V_{AD}}\rangle }{\langle W⊙W\rangle -{\langle W\rangle }^{2}}$$

where ⊙ indicates the Hadamard product, which numerically resulted into *G* = 3.362 ± 0.002 V/kg.

Recalling that the vector *W* is composed of *M* = 20 test weights and that the vector $\bar{V_{AD}}$ is obtained calculating the mean of 20 readings for each input weight, the estimate of the gain uncertainty can be computed as:

(5) $$u\left(G\right)=\sqrt{\sum_{i=1}^{M}{\left(\frac{\partial G}{\partial \bar{V_{AD,i}}}\;u_{C}\left(V_{AD,i}\right)\right)}^{2}}$$

**Figure 10:**
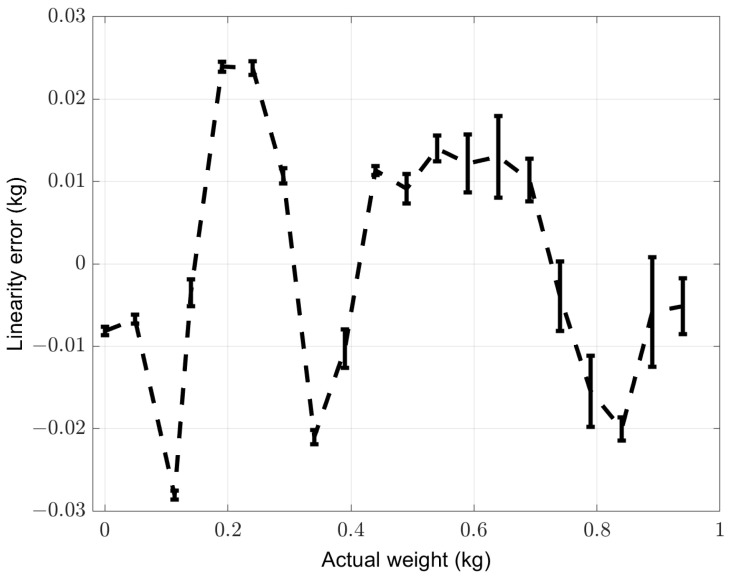
The linearity error of the sensor analog front-end. Error bars represent the uncertainty on linearity error.

Using the gain calculated in Equation (4), we evaluated the deviation from linear regression. In Figure 10, we show the linearity error expressed in kilograms, where error bars represent the uncertainty calculated as described in Equation (3).

From Figure 10, it is possible to see that the maximum nonlinearity of the sensor analog front-end is ≈25 g. This nonlinearity is mainly due to the low-cost pressure sensors we chose, and it is acceptable for our application.

In addition to linearity, repeatability is an important quantity to take into account when using low-cost sensors. To evaluate the repeatability of the sensor analog front-end, we placed and removed 200 times the weight from the sensor and evaluated the standard deviation of the acquired voltages. We chose three significant working points, namely 25%, 50%, and 75% of the full scale range. The corresponding standard deviation resulted into 31 g, 39 g, and 53 g, respectively.

**Figure 11:**
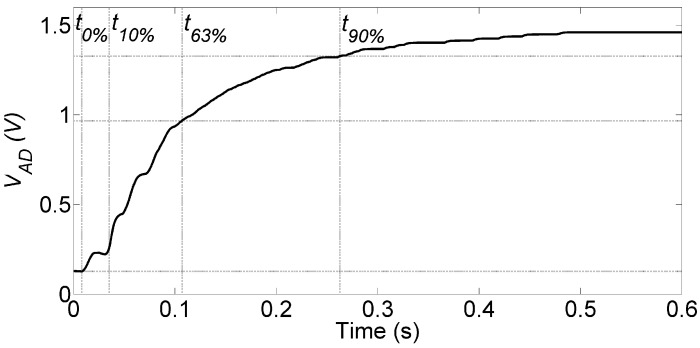
Step response of the subsystem sensor analog front-end. Lines indicate the $V_{AD}$ crossing the 0%, 10%, 63%, and 90% values of its asymptotic range.

The bandwidth of the system was also estimated using the step response method, assuming the dominant pole approximation. A 400 g weight was dropped on the sensor, and the $V_{AD}$ was acquired with the oscilloscope. Figure 11 shows the step response, where in particular we marked the instants $t_{0\%}$, $t_{10\%}$, $t_{63\%}$, and $t_{90\%}$, representing the 0%, 10%, 63%, and 90% of $V_{AD}$, respectively. The bandwidth *B* was then calculated in two ways, namely as $B=0.35/(t_{90\%}-t_{10\%})$ = 1.54 Hz, and using the 63% of excursion $B=1/2\pi (t_{63\%}-t_{0})$ = 1.57 Hz, the two estimates providing coherent results. Since the electronic front-end has a bandwidth of 5 Hz, we can estimate that the mechanical bandwidth of the pressure sensor is approximately 1.5 Hz.

### 3.2. Classifications Results

**Figure 12:**
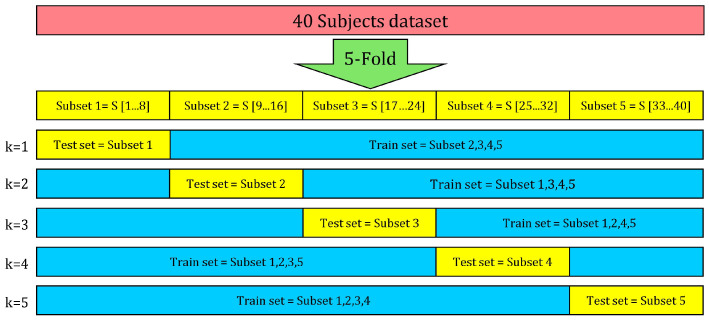
The structure of the 5-fold cross-validation procedure for train and test set splits of the 40 subject dataset.

This paper is mainly focused on subject-independent case, which enforces that the model has to be trained on the data obtained from the subjects not included in the test dataset. Generally speaking, to evaluate a model in case of large datasets, the train/test split is a reliable approach to separate the data [44], while for small to medium size datasets, k-fold cross validation [45] is the most common method [31], together with its special case, the leave-p-out/leave-one-out one [46]. The k-fold cross validation is based on repeating training and testing applied on k-split, not overlapping, equal sizes, subsets of the original dataset [31]. As shown in Figure 12, we implemented a five-fold cross-validation procedure by splitting the experiment dataset (40 subject data) into 5 subsets of the data of 8 subjects each (20%). Each model was trained and tested five times. At each time, four subsets were included in turn in the train dataset, while the remaining one was used as the test dataset. The average accuracy of the five repetitions was then calculated, thus determining the accuracy of the model in the subject-independent case. In this way, each subject data were used exactly once to evaluate each model.

The main metric used to evaluate each classification model was the accuracy [47], defined as the percentage of correct predictions in the test dataset. It was calculated dividing the number of correct predictions by the number of total predictions [48]. Denoting with TP and TN the true positive and true negative predictions, while with FP and FN the false positive and false negative ones, respectively, we can express the accuracy as:

(6) $$Acc.=\frac{TP+TN}{TP+TN+FP+FN}$$

Table 1 shows the accuracy results of the eight postures classification for the seven deep learning models considered, evaluated by the five-fold cross validation described above. The k values represent the subsets used as test dataset. For example, for k = 1 the data of the four [2–5] subsets (subjects [9…40]) populated the train dataset, and the data of the first subset (subjects [1…8]) populated the test one, and so on. The average accuracy of each model calculated from five-fold repetition can be found in the last line of the table.

Table 1 shows that the best classification accuracy was achieved by the EMN model in the k = 2, 4 cases, and by the MLP model in k = 1, 3, 5 cases. The top score average accuracy of 91.68% was achieved by the EMN model. The MLP model, instead, achieved the second best average accuracy of 90.83%. This result is not surprising and also could be expected, since RNN models are indeed purposely designed to extract features from time series thanks to the presence of their internal memory. However, the MLP model scored respectable results in spite of its simpler structure. The CNN model instead, scored the lowest accuracy of 86.99%. The reason can be ascribed to the fact that CNNs are devised primarily to feature extraction in image processing applications. Nevertheless, the combination of the CNN with an ESN, as it is in the EMN model, produced notable results. From Table 1 the average accuracy of all seven models was computed (89.47 ± 1.53%), indicating that the designed sensors system shows high performance with various types of DL models.

**Figure 13:**
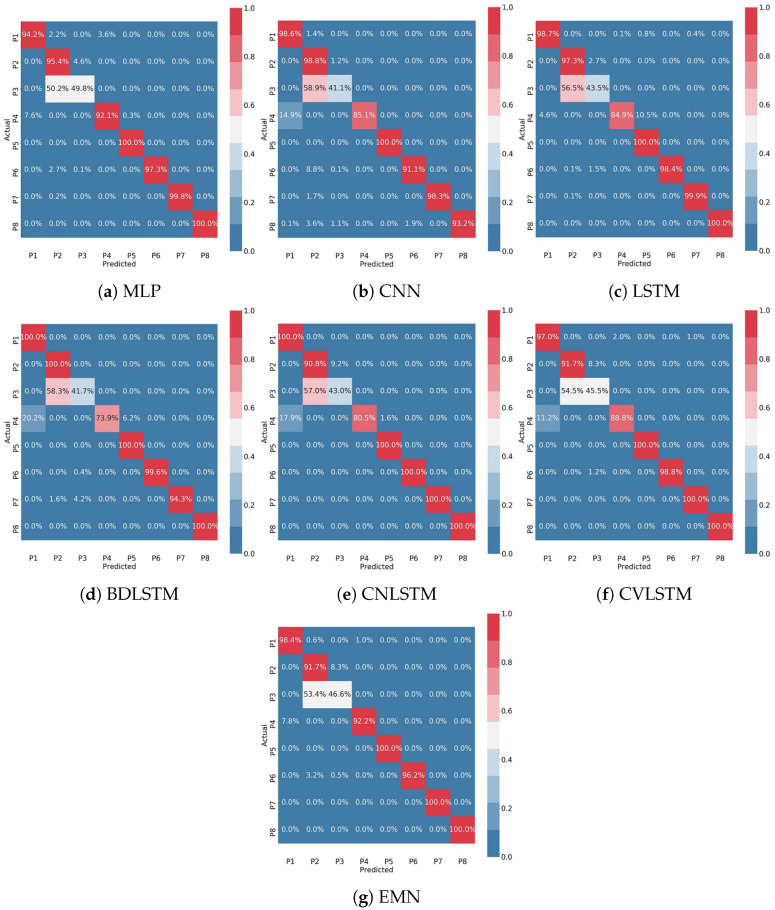
Confusion matrices of 8 postures classification results for 7 DL models, evaluated by 5-fold cross validation. All CMs show the result of k = 1 validation.

The Confusion Matrices (CMs) for all models and for k = 1 are shown in Figure 13. Each row of the confusion matrix represents the actual instances, while each column represents predicted instances. The color intensity of the cells indicates the accuracy level of the predictions, where intense red represents the highest accuracy (90–100%) and intense blue represents lowest accuracy (0–10%). For example, in the case of MLP, as shown in Figure 13a, 49.8% of actual instances of P3 cases were predicted correctly on the P3 category, while 50.2% were predicted incorrectly on the P2 category. All other postures were predicted correctly with an accuracy rate higher than 90%. In the case of the CNN model, Figure 13b, only 41.1% of actual instances of P3 cases were predicted correctly on the P3 category, while 58.9% were predicted incorrectly on the P2 category. CNN could correctly predict the 85.1% of the actual P4 instances as belonging to the P4 category, while it incorrectly predicted the 14.9% of the actual P4 instances as belonging to the P1 category. All other models have similar results, which indicate that it was quite challenging for almost all models to distinguish between P2 and P3, and for most models (CNN, LSTM, BDLSTM, CNLSTM, CVLSTM) to distinguish between P1 and P4. The reason is that P2 and P3 have similar weight distribution on sensors because only in these two postures, generally, none of the backrest sensors are in contact with the user’s body, and the lower part of his body has symmetric weight distribution on the sitting cushion sensors. In addition, in the case of P1 and P4, a similar weight distribution on all sensors could be the reason for the models errors, since these two postures are the only ones with all sensors in contact with the user’s body. Moreover, the chair in our experiment had a rigid backrest, that is another reason for P1 and P4 to show similar weight distribution on both the backrest and sitting cushion sensors.

**Figure 14:**
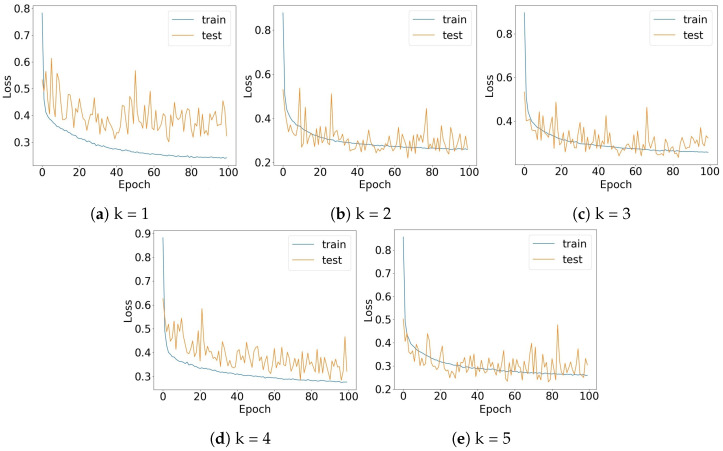
Training and testing loss of the EMN model in all the 5-fold cross validation along 100 epochs. Orange lines show the test losses while the blue lines show the training losses.

Figure 14 shows the training and testing loss of the EMN model for five-fold cross validation. As mentioned in Section 2.3, to prevent overfitting we used a regularization parameter (L2 penalty) in the dense layer for all models, including EMN. Thanks to the architecture of our models, the optimized hyperparameters and the adequate number of training samples, we prevented underfitting as well. Therefore, as suggested by the loss plots of Figure 14, we achieved an acceptable Bias–Variance trade off [49] which resulted in a high accuracy.

## 4. Discussion

In this section, we discuss the different sitting patterns with related classification challenges for deep learning classifiers, the computational costs of the models, and finally a comparison between our best result and those from similar studies.

### 4.1. Sitting Patterns

**Figure 15:**
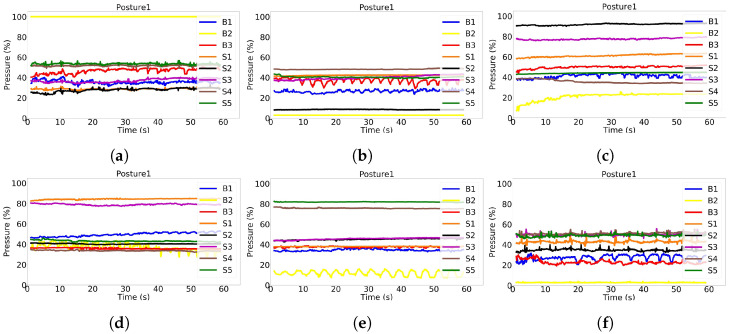
The raw signals of the pressure sensors as obtained from six different subjects sitting in Posture 1 are shown in (**a**–**f**). The weight distributions of the subjects on sensors have different patterns, challenging the models in recognizing them as the same posture.

During the experiment, we noticed that different subjects had different sitting patterns for the same posture. For example, Figure 15 shows the plots of all eight sensors signals during a 60 s period of the P1 posture (sitting upright) for six different subjects. In this posture, the individual in Figure 15a produced the maximum pressure on Back2 sensor, which is positioned on the top middle part of the backrest, and medium pressure on Back1 and Back3 sensors, which are positioned on the top right and left side of the backrest, respectively, a condition that is expected to occur generally in P4 where the subject is bending backwards. Subjects in Figure 15b,e,f, sitting in the P1 posture, were not touching—or slightly touching—the Back2 sensor, but pressing with medium pressure Back1 and Back3 sensors. Subjects in Figure 15c,d, sitting in the P1 posture, were touching with medium pressure Back1, Back2, and Back3 sensors. As far as the sitting cushion sensors are concerned, we noticed that in the case of tall subjects, such as the one in Figure 15d, Seat4 and Seat5 sensors, which are placed on the cushion front right and left sides, respectively, showed lower pressure since the legs’ weight was discharging mostly on the floor. In the case of short subjects, such as the one in Figure 15e, instead, the feet barely touched the floor, and the legs’ weight was mostly discharged on the frontal part of the chair, thus making Seat4 and Seat5 sensors detect high pressure. In the case of a subject with medium height, instead, all of the five seat sensors would detect similar medium pressure (see Figure 15f). Finally, Seat2 sensor, which is positioned in the middle back side of the cushion, in P1 posture was pressed according to the shape of the lower part of the subject’s body and depending on how much the subject was occupying the whole cushion.

**Figure 16:**
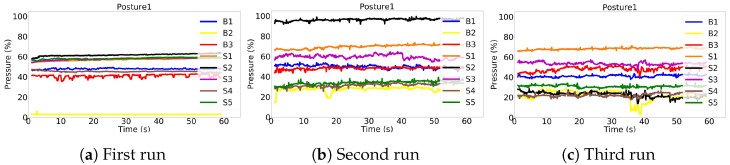
Pressure sensors raw signals during three repetitions of the same subject sitting in Posture 1. The weight distributions of the subject on sensors have different patterns in each repetition, challenging the models in recognizing them as the same posture.

Surprisingly, we noticed that the same subjects could sit quite differently in the same posture during the various repetitions. Figure 16 shows the same subject sitting three times in P1 posture. It can be observed that the weight distribution in each repetition is different, and in the case of Seat2 sensor, the variation ranges from 100% in Figure 16b to 25% in Figure 16c.

**Figure 17:**
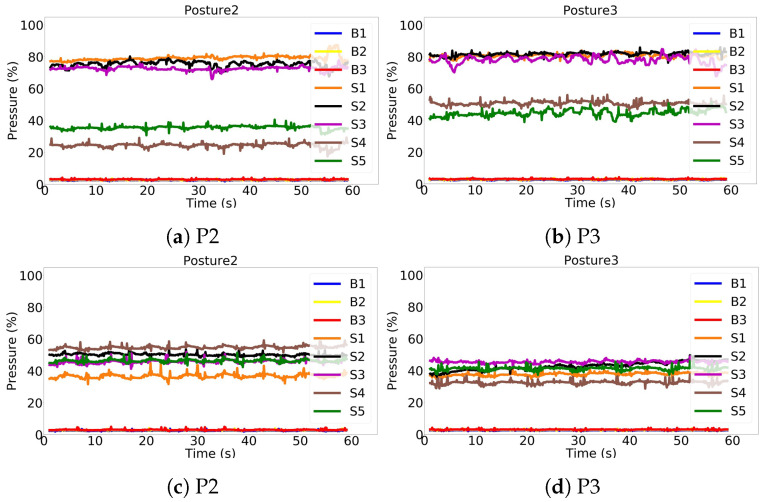
Posture 2 and Posture 3 of different subjects can have similar patterns: (**a**,**c**) are pressure sensors raw signals of two different subjects while sitting in Posture 2. They are similar to (**b**,**d**), respectively, which are pressure sensors raw signals of other two subjects while sitting in Posture 3.

As mentioned in Section 3.2 and shown in CMs, Figure 13, P2 and P3 postures could show similar weight distribution on sensors independently of the participant, thus challenging the models to distinguish between the two. Figure 17 shows the similarity between these postures. Figure 17a shows the weight distribution of a specific subject sitting in posture P2. Figure 17b shows the weight distribution of another subject sitting in posture P3; it can be seen, the two weight distributions are very similar. Again, the P2 weight distribution of the subject in Figure 17c has a similar pattern to the weight distribution of another participant sitting in the P3 posture, as shown in Figure 17d.

Furthermore, we noticed that the pressure applied on each sensor was also varying if the subjects were sitting in the center of the chair or not and depending on how they could keep an upright posture with their back. Some subjects showed the tendency to bend their back to the right, to the left, or forward, moving the center of gravity accordingly. Consequently, the weight distributions recorded on the sensors for these subjects were different from those subjects who sat straight. Other aspects were recognized to influence the sensor readouts, such as body alignment, body shape, mass distribution, height, limbs proportion, and emotional state, such as stressed or relaxed.

### 4.2. Computational Cost

Table 2 shows the computational cost of each model in terms of time and training parameters obtained during the first fold of the five-fold procedure. The first row lists the computational time (in minutes) required to run 100 training epochs on the models and shows that MLP and CNN models had the best efficiency, while BDLSTM and EMN models had the worst, respectively. The second row lists the total trainable parameters of each model. MLP, with its 480 parameters, had the lowest trainable parameters number, while BDLSTM, with its 416,208, had the highest one. The table shows only the first fold of the validation process, since the training parameters were the same for all folds, and the computational time had negligible variation along the whole procedure.

Table 2 suggests that although with better scores, RNN models are more complex and show high computational costs. Consequently, since the MLP model achieved a good score (90.83%) at a relatively low computational cost, it was chosen as a reasonable trade-off for our application.

### 4.3. Study Comparison

As said in Section 1, we compared our designed sensors system with the similar studies that we mentioned there. In Table 3 a more detailed comparison between our design and similar studies with subject-independent cases is shown. The comparison is made in terms of sensor types and numbers, acquisition system, number of postures, number of subjects, employed algorithms, validation technique, and achieved accuracy.

Table 3 suggests that high accuracy was generally achieved by using a high number of sensors, as in [20] where a 92.2% of accuracy was achieved using an array of 8 × 8 sensors, or by a combination of different types of sensors, as in [25] where a 92% accuracy was achieved using the combination of six FSR and six infrared sensors. Furthermore, it can be observed that a higher number of postures under test may result in lower classification accuracy even with a high number of sensors, as in [19]. In any case, the postures that produce similar sensor readouts could also make the classification challenging when fewer postures were tested, as in the case of P2 and P3 of the present study, which had similar weight distribution on the sensors, thus challenging the prediction models in distinguishing them from one another, as explained in detail in Section 3.2 and Section 4.1.

The validation method is very important for the classification result evaluation. In the present study, we computed the average accuracy by means of the k-fold cross-validation method, while some other studies did not mention the validation strategy with which they obtained their results, [19,20,23]. Another factor that could influence the result is the number of subjects participating in the experiment. We conducted a relatively large experiment, involving 40 subject of various ages and body shapes that helped our training models behave rather well in the generalization and show acceptable Bias–Variance trade-offs, as explained in detail in Section 3.2. As mentioned in Section 1, experiments with fewer subject such as [23] may feature high variance.

Moreover, despite the posture detection challenges described in Section 4.1, the designed sensors system achieved a classification accuracy of **91.68%** with the EMN model, which is close to the highest accuracy of Table 3, still using the lowest number of sensors. This relatively high accuracy was achieved thanks to several advantages of the system itself, such as: (1) the sensors were placed directly on the sitting cushion and backrest of the chair and were in direct contact with the user’s body; this condition provided accurate information about the body’s weight distribution on the chair, since interposing a layer in between could damp the pressure; (2) the number of the sensors used on the chair was optimized to provide enough information on all chosen postures; (3) the position of each sensor was chosen to enhance the accuracy of the postures classification algorithms; and (4) the chair was stable and robust, and the sensors were strongly attached to the chair, allowing for accurate and repeatable measurements.

The system also benefits from being simple and easy to setup, from being self-contained as far as power supply and communication are concerned, and since it does not require initialization or calibration procedures at each data acquisition.

## 5. Conclusions

In this research study, a smart chair sensors system was designed, realized, and tested with an experiment involving 40 subjects. A large dataset was created with the acquired data. The performance of the designed sensors system was evaluated with seven deep learning models for eight sitting postures classification and secured by k-fold cross validation. Results of all DL models were compared, and the best average accuracy of 91.68% was achieved by an EMN model, obtained in 5-fold, each fold lasting 27 min for computations and to train the 162,248 trainable parameters. The MLP model, instead, achieved the average accuracy of 90.83%, obtained in 5-fold, each fold lasted 3 min for computations and to train 480 trainable parameters. This second one can be considered an appropriate trade-off model for our application in terms of computational cost vs. accuracy.

The sensors system that was designed is innovative, at the same time, simple and versatile. This was obtained thanks to rather low number of deployed pressure sensors, to the optimization of their position, and to the purposely developed PCB. Furthermore, the adopted design is easily applicable to many types of chair and armchair. The signal acquisition board transmits the data via Wi-Fi to the computer for data acquisition and storage without the need of initialization or calibration processes, and since the board is battery powered, no cables are required, thus allowing for the easy repositioning of the chair, while 30 h of continuous operation is at the same time guaranteed with no need of battery recharging. The system can be employed for various applications, such as emotional, behavior and activity identification. Its design is easily extendable to the case of multiple smart chairs to be operated simultaneously and synchronously for larger postures classification experiments.

## Figures and Tables

**Table 1:** The average classification accuracy (in %) for the eight postures and for the seven DL models as obtained by means of the 5-fold cross validation. The highest accuracy values are shown in bold slanted font.

**k**	**MLP**	**CNN**	**LSTM**	**BDLSTM**	**CNLSTM**	**CVLSTM**	**EMN**
1	***91.07***	88.28	90.33	88.61	89.23	90.16	90.64
2	92.75	91.05	90.7	91.27	91.68	90.84	***94.55***
3	***93.7***	90.41	91.79	91	91.26	91.7	93.51
4	87.14	84.21	84.24	86.44	85.25	87.67	***90.44***
5	***89.47***	81.02	86.45	87.57	88.26	89.49	89.26
*AVG. Acc.*	90.83	86.99	88.71	88.98	89.14	89.97	***91.68***

**Table 2:** The computational time required for the 100 training epochs. Number of trained parameters of the different DL models as obtained in the first fold of the 5-fold cross validation.

**k = 1**	**MLP**	**CNN**	**LSTM**	**BDLSTM**	**CNLSTM**	**CVLSTM**	**EMN**
Minutes	3	4	20	28	9	9	27
Parameters	480	2598	209,008	416,208	267,760	95,760	162,248

**Table 3:** The comparison between our approach and other methods and systems as published in the literature on sitting postures classification.

Study	Sensors Type	No. Sensors	Acquisition System	No. Postures	No. Subjects	Algorithm	Validation	Accuracy (%)
[16]	Tekscan BPMS 42 × 48	31	Tekscan	10	52	SVM	10-fold	87
[19]	Tekscan BPMS 42 × 48	42 × 48	Tekscan	14	30	PCA	-	79
[20]	FSR array 54 × 44	8 × 8	-	8	16	ANN	-	92.2
[23]	16 × 16 textile sensor array	16 × 16	Arduino	7	10	Optimization algorithm	-	79
[25]	FSR & Infrared	12	-	11	36	KNN	10-fold	92
Present study	FSR	8	Custom designed system	8	40	EMN	5-fold	91.68

Legend: SVM: Support Vector Machines; PCA: Principal Component Analysis; ANN: Artificial Neural Network; KNN: K-Nearest Neighbor; EMN: Echo Memory Network.

## Data Availability

Data are available upon request.

## Abbreviations

The following abbreviations are used in this manuscript:

ADC	Analog To Digital Converter
AI	Artificial Intelligence
ANN	Artificial Neural Network
BDLSTM	Bidirectional LSTM
CNLSTM	CNN LSTM
CNN	Convolutional Neural Network
CVLSTM	Convolutional LSTM
DL	Deep Learning
DSP	Digital Signal Processor
EMN	Echo Memory Network
ESN	Echo State Network
FSR	Force Sensing Resistors
GUI	Graphical User Interface
GUM	Guide to the expression of Uncertainty in Measurement
IoT	Internet of Things
KNN	K-Nearest neighbors
LiPo	Lithium-Polymer
LSTM	Long Short Term Memory
MIPS	Million Instructions Per Second
MLP	Multilayer Perceptron
PCA	Principle Component Analysis
PCB	Printed Circuit Board
ReLU	Rectified Linear Unit
RNN	Recurrent Neural Network
TCP	Transmission Control Protocol
UART	Universal Asynchronous Receiver Transmitter
